# Exploring the Molecular and Genomic Landscape of the Dark Agouti Rat Mammary Adenocarcinoma: Preliminary Insights for Triple-Negative Breast Cancer Modeling

**DOI:** 10.1007/s10911-026-09606-x

**Published:** 2026-04-10

**Authors:** Ifeoma J. Dikeocha, Adrien Oliva, Emma Bateman, Hannah R. Wardill, Joanne M. Bowen

**Affiliations:** 1 School of Pharmacy and Biomedical sciences, College of Health, Adelaide University, Level 2 Helen Mayo South, North Terrace, Adelaide, SA 5000 Australia; 2 Australian e-Health Research Centre, Commonwealth Scientific and Industrial Research Organization (CSIRO), Melbourne, 3169 Australia; 3 Supportive Oncology Research Group, Precision Cancer Medicine, The South Australian Health and Medical Research Institute, Adelaide, Australia

## Abstract

The isogenic, tumor-bearing Dark Agouti Mammary Adenocarcinoma (DAMA) model is commonly utilized in breast cancer research to evaluate mechanisms of, and interventions for chemotherapy-induced toxicity and tumor response. However, the biological subtype, immune landscape, and genomic profile of DAMA have remained uncharacterized. In this study, we comprehensively evaluated DAMA tumors naïve and exposed to methotrexate, assessing their histopathological, molecular, and genomic features. Our findings reveal that the DAMA model presents a platform to model the human triple-negative breast cancer (TNBC), as it exhibits substantial macrophage infiltration, the tumors display dysregulation of oncogenes *Bcl2*, *Egfr*, and potentially *Myc*, suggesting apoptotic resistance as a key mechanism driving growth. These characteristics position the DAMA model as a potential preclinical model for investigating TNBC biology, therapeutic responses, and drug toxicity.

**Supplementary Information:**

The online version contains supplementary material available at 10.1007/s10911-026-09606-x.

## Introduction

 Breast cancer (BC) remains a significant global health challenge, continuing to account for a substantial proportion of cancer-related mortality. Effective management increasingly relies on the implementation of sophisticated, personalized treatment regimens tailored to the molecular and pathological characteristics of individual tumors [1]. Breast cancer is classified into intrinsic molecular subtypes originally defined by gene expression profiling, which reflect distinct biological characteristics and prognostic outcomes. In clinical practice, surrogate markers such as hormone receptor status and proliferation indices are commonly used to approximate these subtypes (Luminal A, Luminal B, HER2-enriched tumors, Triple-negative breast cancer and Normal-like tumors) that differ in prognosis and therapeutic response, shaping modern precision treatment strategies [2–6].

Rodent models have long been essential to cancer research, aiding in the identification of carcinogens, evaluation of preventive agents, and therapeutic screening. Various breast cancer models, including spontaneous, induced, and transgenic tumors in wild-type and knockout mice or rats, have advanced our understanding of disease mechanisms [7].

The Dark Agouti mammary adenocarcinoma (DAMA) is a spontaneously arising tumor from the DA strain that has been adapted as a transplantable, syngeneic, non‑metastatic model with reproducible growth kinetics, enabling controlled experiments in immunocompetent rats. This model has been used extensively to study chemotherapy‑induced toxicity, particularly gastrointestinal mucositis triggered by agents such as methotrexate, 5‑fluorouracil, and irinotecan [8–11], and has underpinned methodological advances including validated readouts like plasma citrulline, diarrhea scoring, histopathological injury indices, and welfare metrics (e.g., activity and body‑composition) for mechanistic and interventional research [12, 13]. Beyond toxicity modeling, DAMA is a well‑established platform for supportive oncology and nutritional intervention studies [14, 15]. Dietary strategies most notably extensively hydrolyzed whey protein combined with medium‑chain triglycerides have shown to attenuate GI mucositis, reduce diarrhea and weight loss, increase microbial diversity/resilience, and preserve chemotherapy efficacy against DAMA tumors [16], highlighting the model’s translational utility for testing gut‑targeted supportive care without compromising anti‑tumor activity. Collectively, the DAMA model’s combination of predictable tumor growth, robust toxicity and welfare endpoints has made it a cornerstone for studying mechanisms of chemotherapy injury, microbiota‑toxicity interactions, and nutritional/supportive interventions in a breast cancer context.

Despite its extensive use, the biological or molecular subtype and immune composition of the DA rat mammary adenocarcinoma remains undefined. In addition, it is currently unknown if mutational burden in DAMA is affected by exposure to chemotherapy. This gap in knowledge is critical, as biological subtypes, each with unique therapeutic responses [17–19] and immune composition need to be considered in preclinical models of chemotherapy treatment and resistance.

This study aimed to comprehensively characterize DAMA and determine its corresponding breast cancer subtype. Through this characterization, we seek to enhance the utility of DAMA as a preclinical model by improving its relevance and applicability in breast cancer research. We investigated the tumor’s immune microenvironment, assessed cellular morphology, and identified genomic variants commonly associated with human breast cancer that inform therapeutic decision-making.

## Materials and Methods

### Tumor Samples

Tumors analyzed in this study originated from a single donor lineage: cells from the initial inoculum were passaged once before implantation into experimental rats. Six DA rats aged 6–8 weeks received subcutaneous injections of 0.2 ml tumor inoculum (2.0 × 10^7^ cells per ml in PBS). These cells developed into a tumor, which was subsequently transferred from the passage rats to experimental rats as previously described in detail [20]. The tumors from the passage rats were homogenized, washed and resuspended in sterile saline, then 2 × 10^6^ DAMA cells were implanted subcutaneously into the left and right flank of the experimental rats that received methotrexate (MTX) at a dose of 0.7 mg/kg for 4 cycles (MTX treated) [14]. The MTX stock concentration of 25 mg/ml diluted in saline was administered intramuscularly. After euthanasia, only right flank tumor samples MTX naïve (*n* = 2), MTX treated (*n* = 4) were snap frozen for molecular assessment and fixed for morphological assessment. Tumors were considered independent units as each tumor analyzed was from six different rats, acknowledging shared origin from a common inoculum.

### Histopathology

#### Hematoxylin and Eosin Staining

Tumors were fixed in 10% neutral buffered formalin, paraffin embedded, and 5µ sections cut and stained with hematoxylin and eosin (H&E). All slides were scanned using Nanozoomer Digital Slide Scanner and viewed using the Nanozoomer Digital Pathology Software (NDP View v2.0, Histalim). 

#### Immunohistochemistry (IHC)

Paraffin-embedded DAMA tumor Sect. (4 μm) were prepared using a rotary microtome (Leica, Germany). Slides were dewaxed, rehydrated, and subjected to heat-induced antigen retrieval in citrate (pH 6.0) or Tris/EDTA (pH 9.0) buffers depending on the primary antibody (Table 1). staining was performed using Dako reagents and an automated system (AutostainerPlus, Dako; AS480). Blocking steps included peroxidase (10 min) and protein inhibition (30 min). Sections were incubated with primary antibodies (60 min), followed by HRP-conjugated secondary detection and DAB visualization. Counterstaining was performed with Harris hematoxylin, followed by differentiation, dehydration, and xylene clearing. Slides were mounted with Entellan mounting media and scanned using the Nanozoomer system (Hamamatsu Photonics). The staining patterns and intensities for each of the antibody were viewed and assessed using the Nanozoomer Digital Pathology software view.2 program (NDP View v2.0, Histalim).

**Table 1:** Primary Antibody Details

Antibody (Manufactured by Abcam)	Dilution used	Antigen retrieval buffer
Recombinant anti-ErbB2 / HER2 antibody (ab214275)	1/3000	Tris/EDTA buffer (pH 9.0)
Anti-Estrogen Receptor alpha antibody (ab3575)	1/5000	Tris/EDTA buffer (pH 9.0)
Recombinant anti-Progesterone Receptor antibody (ab101688)	1/400	Citrate buffer (pH 6.0)
Recombinant anti-Ki67 antibody (ab16667)	1/200	Citrate buffer (pH 6.0)

### Identification of Molecular Subtypes

Tumors were assessed by I.J.D and J.F. Tumors with ≥1% nuclear-stained cells were considered positive for ER and PR expression [21]. Luminal A and B subtypes were distinguished using a 14% cut-off for nuclear Ki-67 expression [22]. HER2 staining was scored based on American Society of Clinical Oncology and College of American Pathologists guidelines: 0 (no staining), 1+ (weak incomplete staining), 2+ (complete non-uniform or weak staining in ≥ 10% of cells), and 3+ (uniform intense staining in > 30% of cells) [23]. Based on IHC findings of ER, PR, HER2, and Ki-67 expression, in human studies, breast cancer is divided into four subtypes: luminal A (ER + and/or PR +, HER2 - and Ki-67 < 14%); luminal B (ER + and/or PR +, HER2 - and Ki-67 ≥ 14% or ER + and/or PR + and HER2 + irrespective of Ki-67 expression); HER2-enriched (ER -, PR - and HER2 +); and triple-negative breast cancer (TNBC) (ER -, PR - and HER2 -) [24].

### Detection of Immune Cell Presence in Tumor

The same IHC protocol described above was used to detect immune cell presence in the DAMA tumor using Anti-CD11b antibody (ab133357, 1:6000 dilution) and Anti-CD45 antibody (ab10558, 1:200 dilution). Scoring of tumors was done by I.D based on visual intensity of the stain characterized as negative staining = 0, weak staining = 1(+), medium staining = 2 (++) strong staining = 3(+++) [25].

### Genomic Characterization of DAMA Tumor

For genomic characterization of DAMA, frozen tumor samples MTX naïve (*n* = 2), MTX treated (*n* = 4) were sent to the Australian Genome Research Facility (AGRF) for DNA extraction, whole genome sequencing and bioinformatic analysis.

DNA extraction from tumors was performed on 20 mg samples using a DNeasy Blood and Tissue kit according to manufacturer’s instructions. Library preparation used the Illumina DNA Prep PCR-Free library preparation method, and sequencing was performed to a depth of > 100 Gbp using an Illumina NovaSeqX-Plus system fitted with a 10B flow cell in 2 × 150 PE configuration. Primary analysis was performed in real time by the NovaSeq Control Software (NCS) v1.2.2.48004 and Real Time Analysis (RTA) v4.6.7. RTA performed real-time base calling on the NovaSeq instrument computer while the Illumina DRAGEN BCL Convert 07.021.645.4.0.3 pipeline was used to generate the sequence data. Afterwards, Ensembl’s Variant Effect Predictor (VEP), (v105.0) [26] was used to annotate the hard.filtered.vcf.gz file, in the case of SNVs and indels, and the sv.vcf file, in the case of structural variants. VEP provides location annotations, such as gene and exonic/intronic; consequence annotations and such as amino acid change, as well as damaging predictions, such as SIFT. The annotation was based on the VEP RefSeq cache for Rattus norvegicus (assembly mRatBN7.2), version 105. In lieu of matched non‑tumor DNA, we removed strain polymorphisms by subtracting DA/OlaHsd germline variants from the Hybrid Rat Diversity Panel (HRDP; PMCID: PMC11880076) using BCFtools v1.20, ensuring cross‑animal “consistent” calls did not reflect strain background. All analyses used mRatBN7.2 (rn7); functional consequences were assigned with Ensembl VEP v105 (RefSeq‑preferred) mRatBN7.2 was the best‑supported rat assembly during these analyses. From VEP‑annotated VCFs, we focused on variants within ± 50 kb of cancer‑relevant genes (*Bax*, *Bcl2*, *Esr1*, *Esr2*, *Egfr*, *Erbb2 Erbb*3, *Erbb4*, *Myc*, *Tp53*, *Pgr*, *Pten*, *Kras*) and flagged high Variant allele frequency (VAF) at allele frequencies > 0.35. Structural‑variant (SV) callsets were merged with SURVIVOR v1.0.7; we used split‑read support > 0.35 as a proxy for high VAF, retained only PASS SVs, and excluded recurrent SVs present in > 4 samples.

We analyzed 13 key cancer-related genes: *Bax*, *Bcl2*, Esr*1*, *Esr2*, *Egfr*, *Erbb2*, *Erbb3*, *Erbb4*, *Myc*, *Trp53*, *Pgr*, *Pten*, and *Kras*. These genes were selected based on their established and targetable roles in cancer pathways, including apoptosis regulation (*Bax*,* Bcl2*), hormone signaling (*Esr1*, *Esr2*, *Pgr*), growth factor signaling (*Egfr*, *Erbb2*, *Erbb3*, *Erbb4*), cell cycle control (*Trp53*, *Pten*), and oncogenic activation (*Myc*, *Kras*).

For each gene, specific genomic coordinates were identified, from the reference mRatBN7.2, and variants were extracted from the annotated VCF files using BCF tools with the following command structure:

bcftools view -r [GENE_COORDINATES] [INPUT_VCF] -Oz -o [OUTPUT_VCF].

To facilitate comparison of genetic variants across all samples, the individual VCF files for each gene were merged into a single file using BCF tools merge and then converted to a tabular format for further analysis and visualization. Genotype data was processed to distinguish between no mutation (0/0), heterozygous mutations (0/1, 1/0), and homozygous mutations (1/1) for each variant position in each sample.

Raw WGS data deposited on the National center for Biotechnology Information portal (NCBI BioProject: PRJNA1406328 ).

#### Data Analysis and Visualization

All data analysis and visualization were conducted using Python (v3.8) with the pandas, matplotlib, seaborn, and numpy libraries. To assess the total mutational burden present in each subject, single nucleotide polymorphisms (SNPs) and insertion/deletion mutations (INDELs) were quantified for each sample across the genome. Total counts of SNPs and INDELs were computed from the annotated VCF files and visualized using a custom Python script. A grouped bar chart was constructed to compare SNP and INDEL counts across individual samples. Additionally, pie charts were embedded within the same figure to depict the proportional distribution of SNPs versus INDELs for each sample.

For the targeted gene analysis, mutation frequencies were normalized by gene length to account for the substantial variation in gene sizes among the analyzed targets. Gene lengths were obtained from the reference mRatBN7.2 genome assembly, and mutations were counted for each gene length for each sample. This normalization approach enabled meaningful comparison of mutational burden across genes of varying sizes, from small genes like Kras (3.78 kb) to large genes like Erbb4 (1073.01 kb). The normalized mutation frequencies were visualized as a grouped bar chart showing the relative mutation across all target genes for each individual sample.

### Statistical Analysis

To evaluate potential MTX-related effects on gene mutation frequencies, samples were stratified into two groups: MTX naïve and MTX treated samples. Statistical analysis was performed on normalized mutation frequency data using a complementary multi-approach strategy to account for the small sample size and unknown data distributions.

Data normality was first assessed using the Shapiro-Wilk test for each gene across all samples (α = 0.05). Given the predominance of non-normal distributions and small sample size limitations, the non-parametric Wilcoxon rank-sum test (Mann-Whitney U test) was performed for all genes regardless of distribution normality, as this approach makes no distributional assumptions and maintains robustness with limited sample sizes. Statistical significance was set at α = 0.05 for all tests with p-values corrected for multiple testing using the (Benjamini-Hochberg or BH adjusted) FDR [27].

## Results

**Fig. 1:**
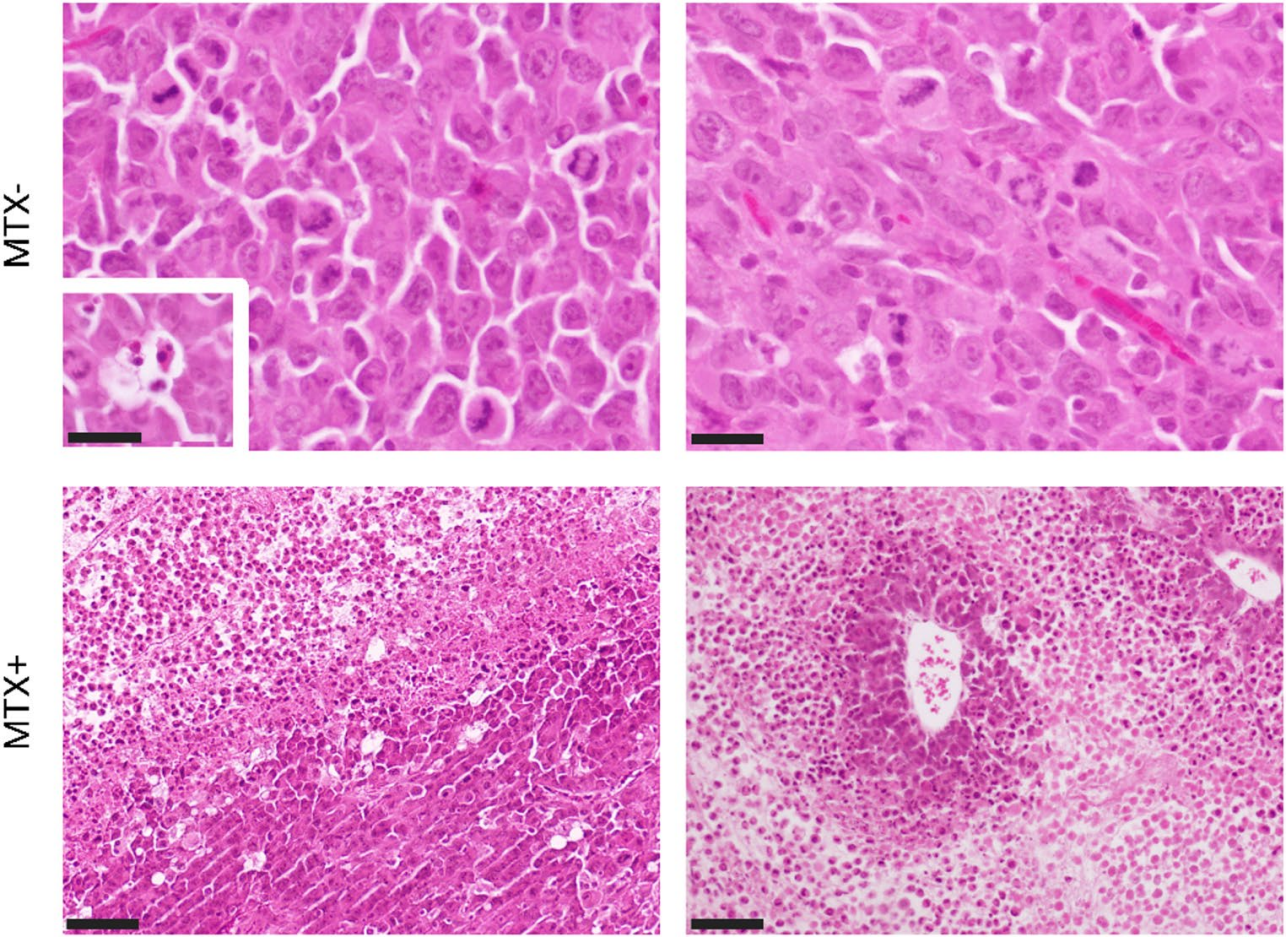
Representative images from H&E stain of DAMA tumors. MTX-: Carcinoma cells of varying size and shape, with karyomegaly and prominent, usually multiple, nucleoli. Numerous mitoses, some abnormal Scale bar − 50 μm. (Inset bottom left − 25 μm) shows an apoptotic body. MTX+: Central MTX induced tumor necrosis, with a viable outer rim of tumor cells, Perivascular rim of more viable tumor cells, surrounded by tumor necrosis. Scale bar − 100 μm. MTX- = MTX naïve, MTX + = MTX treated

**Fig. 2:**
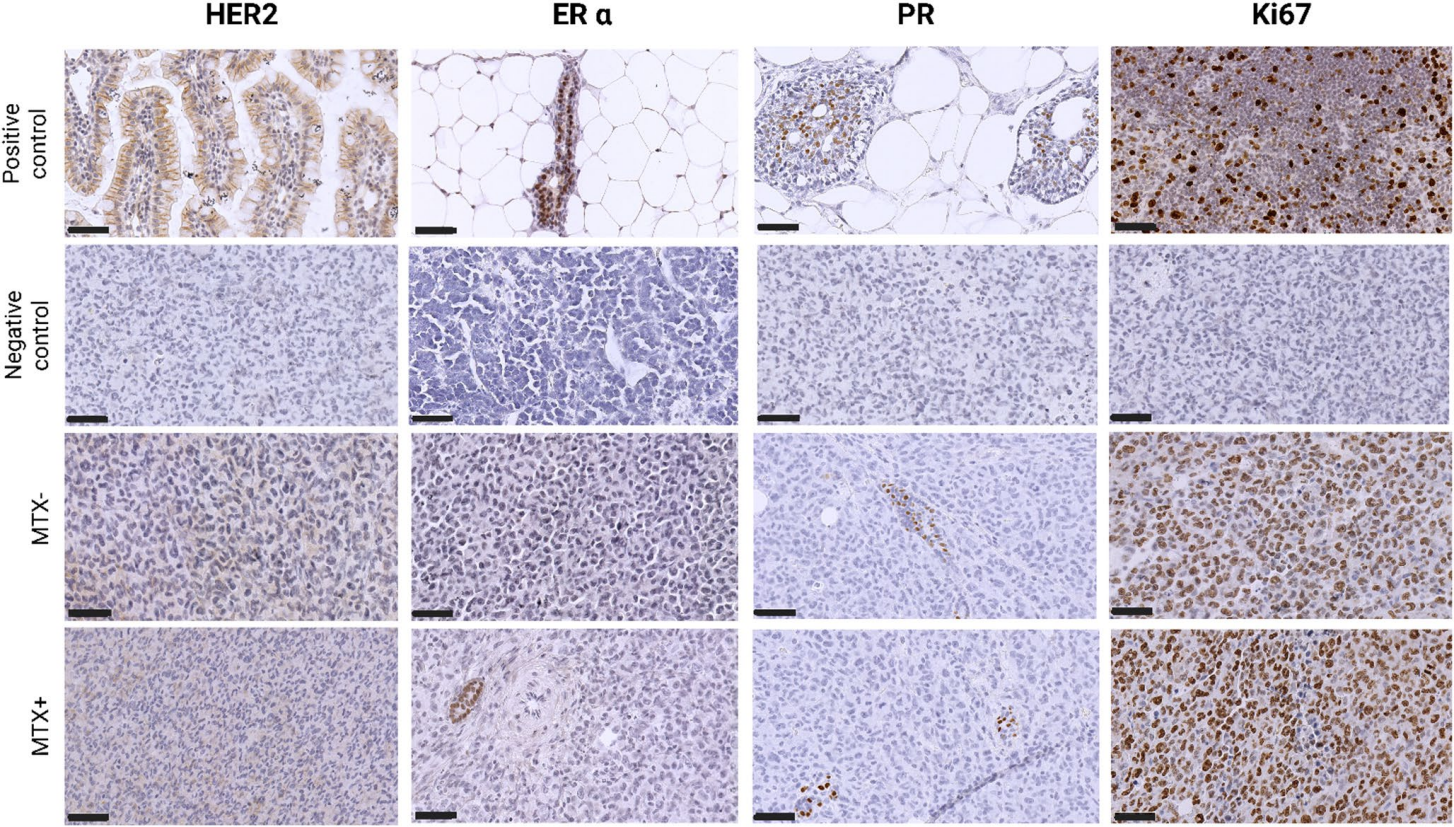
IHC staining for HER2 showing weak membrane staining in DAMA tissue with a degree of intensity observed in < 1% of tumor membrane. ER α and PR proteins showing weak membrane staining in DAMA tissue with < 1% nuclei staining and Ki-67 staining revealed intense positivity in > 14% of cells. MTX- = MTX naïve, MTX + = MTX treated. HER2 positive control = Rat Jejunum, Er α positive control = Rat breast tissue, PR positive control = Rat prostate tissue, Ki67 positive control = Rat spleen, Negative control = DAMA tumor. Scale bar- 50 μm

**Fig. 3:**
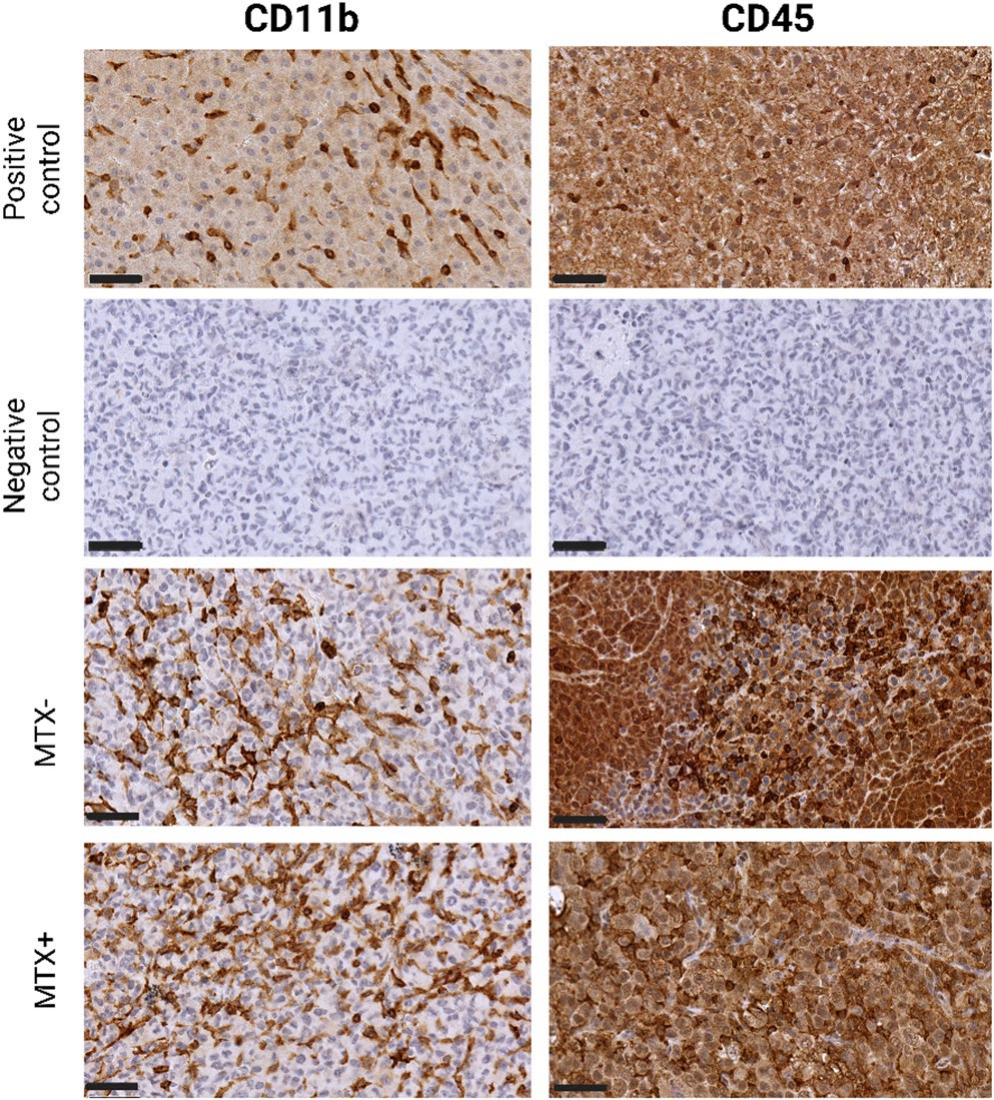
IHC staining for CD11b and CD45 proteins showing macrophages and leukocyte common antigen (infiltrating immune cells) detected in DAMA tumors. MTX- = MTX naïve, MTX + = MTX treated. CD11b positive control = Rat Liver, CD45 positive control = Rat spleen, Negative control = DAMA tumor. Scale bar- 50 μm

Histological and molecular analyses of the DAMA tumor reveal features consistent with a highly aggressive and poorly differentiated tumor. Morphologically, this tumor presented as a solid sheet of closely apposed, poorly differentiated carcinoma cells (Fig. 1), which were round to ovoid in shape, with prominent nucleoli, and a generally high nucleus: cytoplasmic ratio. Mitotic figures were common, many being abnormal, and apoptotic bodies were scattered throughout the tumor. No glandular elements were found, reflecting the lack of differentiation. There was multifocal tumor necrosis, which was greatly increased in MTX-treated tumors, the latter being mostly centrally located in the tumor mass with a peripheral rim of viable tumor cells (Fig. 1). Molecular profiling shows weak membrane staining for the anti- HER2 antibody (scored 1+), signifying low HER2 protein expression across all tumor samples. Additionally, nuclear staining for anti-Estrogen Receptor (ER) alpha and anti- Progesterone Receptor (PR) antibodies was less than 1%, classifying the tumor as negative for hormone receptor expression. In contrast, anti-Ki-67 antibody staining revealed intense positivity in > 14% of cells, suggesting a high proliferative index. These findings collectively support the classification of the DAMA tumor as analogous to the human triple-negative breast cancer (TNBC) subtype, characterized by low HER2, ER, and PR protein expression and elevated Ki-67 proliferation (Fig. 2). Immunohistochemical analysis further revealed medium (2 ++) to strong (3 +++) CD11b expression in the tumor sections, and uniform strong CD45 expression (3 +++), indicating robust immunological activation within the tumor microenvironment, (Fig. 3).

### Bioinformatic Analysis

**Fig. 4:**
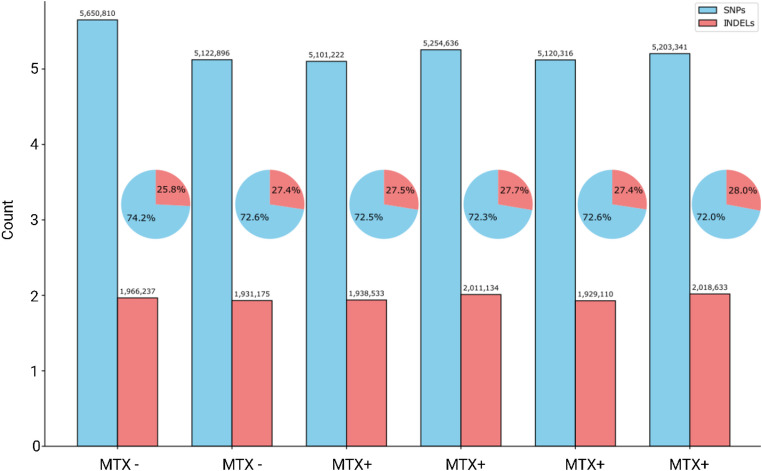
Genomic variant distribution across Dark Agouti mammary adenocarcinoma (DAMA) tumor samples. Bar graph showing total counts of single nucleotide polymorphisms (SNPs, blue) and insertion/deletion mutations (INDELs, red) identified in each sample. Embedded pie charts display the proportional distribution of SNPs versus INDELs for each sample, with percentages indicated

**Fig. 5:**
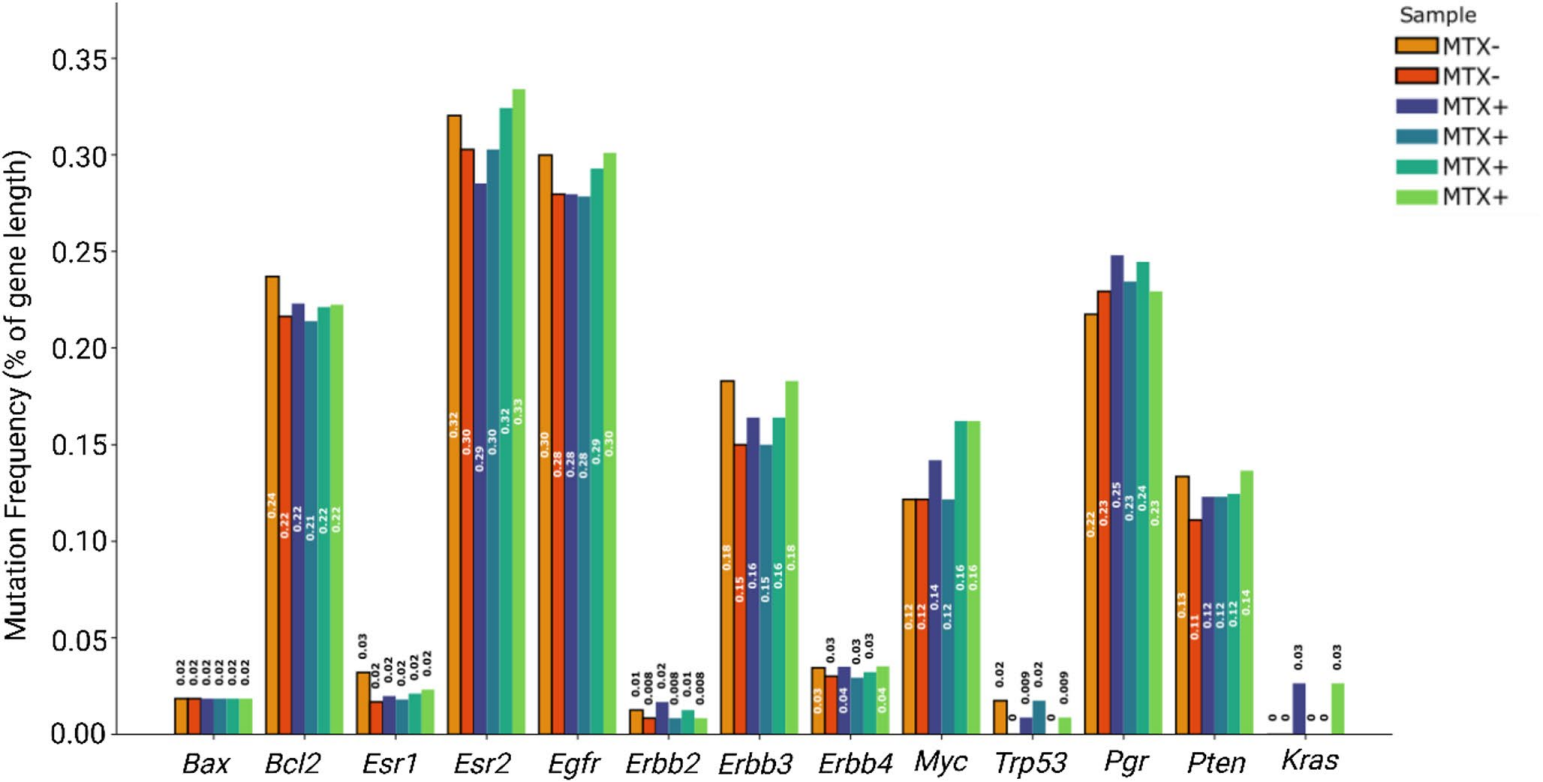
Normalized mutation frequency across 13 cancer-related genes in DAMA tumor samples. Bar graph shows the percentage of mutations detected in each of the analyzed cancer-related genes (*Bax*, *Bcl2*, *Esr1*, *Esr2*, *Egfr*, *Erbb2*, *Erbb3*, *Erbb4*, *Myc*, *Trp53*, *Pgr*, *Pten*, and *Kras*), normalized by gene length, across all six samples. Values shown above each bar represent the mutation frequency as a percentage relative to the length of each gene

Analysis of sequencing data from six DAMA tumor samples revealed considerable genomic variation, with all samples exhibiting substantial numbers of genomic variants. Single nucleotide polymorphisms (SNPs) comprised approximately three-quarters of all variants across samples (Fig. 4 and 5). The total SNP count ranged from 5,101,222 to 5,650,810 across samples. The number of insertion/deletion (INDELs) showed less variation between samples, ranging from 1,929,110 to 2,018,633. The proportion of SNPs to INDELs remained relatively consistent across most samples, with SNPs comprising 72.0-72.6% of variants in five of the six samples with the sixth being 74.2%. After subtracting DA/OlaHsd strain germline variants using the HRDP resource, we observed 1,292 oncogene‑proximal consequences across all tumors, comprising 580 High‑VAF (44.9%) and 712 Low‑VAF (55.1%) calls. The spectrum was overwhelmingly intronic (1,274/1,292; 98.6%), with only six events each annotated as upstream, downstream, or non‑coding transcript exon (Supplementary data Figure 2). Per‑sample totals ranged from 175 to 239, median 217; High‑VAF fractions spanned 40.8–46.9% (Supplementary Table 2). We identified 849 oncogene‑proximal SVs overall, including 627 High‑VAF (73.9%) and 222 Low‑VAF (26.1%) events. The SV landscape was dominated by deletions and duplications (DEL 39.6%, DUP 39.2%), followed by breakends (12.9%) and insertions (8.2%); notably, no Low‑VAF insertions were detected ( Supplementary data Figure 2). Per‑sample SV burdens ranged from 109 to 171, median 147, with consistently high High‑VAF fractions (69.0–78.4%) across samples (Supplementary Table 2).

To better characterize the mutations and genetic landscape of the DAMA tumor model, we performed targeted analysis of 13 breast cancer-related genes associated with key oncogenic pathways, with mutation frequencies normalized by gene length to enable meaningful comparison across genes of varying sizes.

When accounting for gene length, *Esr2* and *Egfr* emerged as the genes with the highest normalized mutation frequencies, with *Esr2* showing mutation rates of approximately 0.28–0.33 mutations per kilobase across samples, and *Egfr* displaying rates of 0.27–0.30 mutations per kilobase. These high normalized frequencies suggest these genes may be particularly susceptible to mutagenic processes or may harbor functionally relevant hotspots in the DAMA tumor model.

*Bcl2*, demonstrated high normalized mutation frequencies of approximately 0.22–0.23 mutations per kilobase across samples, indicating a mutation rate consistent with genome-wide background levels when gene size is considered. Similarly, *Pgr* showed normalized frequencies of 0.21–0.25 mutations per kilobase, suggesting high mutational burden relative to its gene length.

Several genes exhibited notably low normalized mutation frequencies despite their established roles in cancer biology. *Bax*, *Esr1*, *Erbb2*, *Erbb4*, *Trp53*, and *Kras* all showed normalized frequencies below 0.05 mutations per kilobase, with some samples showing zero or minimal mutations in these genes when adjusted for their relatively small sizes. The low mutation burden in these genes, particularly *Trp53* and *Kras* which are frequently mutated in human cancers, suggests that DAMA tumor development may rely on alternative oncogenic mechanisms. *Erbb3* and *Pten* showed intermediate normalized mutation frequencies ranging from 0.12 to 0.18 mutations per kilobase, indicating moderate mutational burden relative to their gene lengths. The consistent mutation patterns across samples for most genes suggest conservation of key genomic alterations in the tumor samples regardless of MTX treatment.

Given inclusion of both MTX naïve (*n* = 2) and MTX-treated samples (*n* = 4) in our dataset, we performed statistical analysis to explore potential treatment-induced changes in gene mutation frequencies. Shapiro-Wilk normality testing revealed that most genes (10 of 13) exhibited non-normal distribution patterns, genes with higher mutation frequencies and greater inter-sample variability (*Bcl2*, *Esr1*, *Esr2*, *Egfr*, *Erbb2*, *Erbb3*, *Pgr*, *Pten*) consistently demonstrated non-normal distributions, while genes with relatively stable, low mutation frequencies showed patterns approaching normality. (Supplementary data Table 1)

Given the predominance of non-normal distributions across the genes, Wilcoxon rank-sum analysis was performed to assess treatment-related changes (Table 2). This non-parametric approach revealed statistically significant differences (*p* < 0.05, FDR adjusted *p* < 0.05) in mutation frequencies for 4 of 13 genes between untreated controls and chemotherapy-treated samples. Specifically, three genes showed significantly increased mutation frequencies following treatment: *Myc*, *Pgr* and *Kras* while one gene demonstrated significant decreased mutation frequencies: *Bax*.

**Table 2:** Wilcoxon Rank-Sum Test Results for All Genes. Includes U Statistics, *p*-values, FDR Adjusted p-values using the Benjamini-Hochberg (BH) Correction, Significance, and Effect Direction for all Genes Regardless of Distribution

Gene	Control Median (Range)	Treated Median (Range)	U Statistic	*P*-value	Significance	Effect Direction	FDR (BH) Adjusted *P*-value
*Bax*	1 (1–1)	1 (1–1)	0.00	0.0071	Significant	Decreased	0.0231
*Bcl2*	372 (355–389)	364 (351–366)	3.00	0.2257	Not significant	Decreased	0.3260
*Esr1*	96 (66–126)	80.5 (71–91)	4.00	0.6886	Not significant	Decreased	0.6886
*Esr2*	159.5 (155–164)	160.5 (146–171)	3.00	0.2537	Not significant	Increased	0.3298
*Egfr*	500.5 (483–518)	494.5 (481–520)	4.00	0.3673	Not significant	Decreased	0.3979
*Erbb2*	2.5 (2–3)	2.5 (2–4)	2.00	0.0407	Not Significant	Decreased	0.1058
*Erbb3*	35.5 (32–39)	35 (32–39)	3.00	0.2823	Not significant	Decreased	0.3336
*Erbb4*	346.5 (323–370)	361 (315–378)	3.00	0.2194	Not significant	Increased	0.3260
*Myc*	6 (6–6)	7.5 (6–8)	0.00	0.0042	Significant	Increased	0.0182
*Trp53*	1 (0–2)	1 (0–2)	3.00	0.1652	Not significant	Decreased	0.3260
*Pgr*	131.5 (128–135)	141 (135–146)	0.00	0.0034	Significant	Increased	0.0182
*Pten*	81.5 (74–89)	82.5 (82–91)	3.00	0.1971	Not significant	Increased	0.3260
*Kras*	0 (0–0)	0.5 (0–1)	0.00	0.0012	Significant	Increased	0.0156

The remaining 9 genes (*Bcl2*, *Esr1*, *Esr2*, *Egfr*, *Erbb2 Erbb3*, *Erbb4*, *Pgr*, *Pten*) showed no statistically significant differences between treatment groups (*p* > 0.05), suggesting either stable mutation profiles or insufficient statistical power to detect treatment effects given the limited sample size, which substantially constrains statistical power and increases the risks of both false negative and false positive findings.

## Discussion

We sought to characterize the previously unclassified biological and molecular subtype, as well as the immune microenvironment, of the Dark Agouti rat mammary adenocarcinoma (DAMA). Furthermore, we investigated whether exposure to chemotherapy influences the tumor’s mutational burden. The tumor subtype classification relied on human TNBC criteria applied to rat tissue without accounting for species-specific differences in antigen expression or staining background. However, we cautiously infer that the DAMA tumor reflects the molecular attributes of triple negative breast cancer (TNBC) a clinically challenging tumor in need of treatment innovation. Hence, this data may position the DAMA model as a potential clinically meaningful platform to better understand and treat TNBC.

TNBC comprises 15–20% of BC cases [28], and is currently treated with chemotherapy, immune checkpoint inhibitors, and targeted agents such as PARP inhibitors [29]; however, prognosis remains poor due to high recurrence rates and limited therapeutic options [30]. Recent preclinical studies have used murine syngeneic models, such as 4T1 and EMT6, to study the mechanisms of TNBC and develop new therapies [31]. However, these models often lack the genetic complexity of human TNBC, grow rapidly, and are limited by species-specific immune responses. Moreover, rat models for TNBC remain underutilized, with most existing models focusing on hormone receptor-positive disease [32]. This highlights the potential of the DAMA model to be utilized more broadly for TNBC in-vivo studies. As a syngeneic tumor model in immunocompetent rats, DAMA enables investigation of tumor-immune interactions and immunotherapy efficacy while offering physiological advantages over murine systems. Its genomic characterization and triple-negative phenotype further support its relevance, potential and translational value in TNBC research.

We observed CD11b and CD45 positivity attributable to the infiltration of immune cells, including lymphocytes and macrophages. These findings suggest that the tumor immune microenvironment of DAMA may be a suitable platform for TNBC, with immunotherapy being a potential therapy class to investigate in the model [33]. Emerging evidence indicates that TNBC exhibits the highest level of immune activation among BC subtypes, as demonstrated by elevated levels of tumor-infiltrating lymphocytes (TILs) and immune-related gene expression within both intratumoral and stromal compartments [34]. Immune checkpoint inhibitors (ICIs), particularly anti-PD-1/PD-L1 antibodies combined with taxanes, have shown promise in treating TNBC [35]. ICIs, including anti-CTLA-4 (ipilimumab), anti-PD-1 (nivolumab, pembrolizumab), and anti-PD-L1 (atezolizumab, durvalumab, avelumab), are in various stages of clinical trials with encouraging early results [36]. According to the latest eviQ guidelines, ICI specifically pembrolizumab are now part of the standard of care for TNBC in certain settings. However, ICI monotherapy often yields limited efficacy, prompting ongoing investigations into combination strategies that target both tumor cells and the tumor microenvironment to enhance therapeutic outcomes [37]. This study is based on CD45 and CD11b staining only, without assessment of effector T cells, activation/exhaustion markers, or spatial localization. Consequently, the tumors may represent a myeloid-dominant or immune-excluded phenotype. Further characterization of DAMA could include exploration of additional antigen-presenting cells and spatial analysis to further delve into the stroma composition which may uncover targets for therapy.

From our genomic sequencing and bioinformatic analysis of the normalized gene mutation frequency in DAMA tumor, the mutational landscape across all tumor samples revealed that the Estrogen receptor beta (*Esr2*) gene, implicated for exerting antiproliferative effects [38] had the highest mutation frequency out of the 13 genes evaluated. Followed by *Egfr* gene based on variant frequency alone, *EGFR* is a major oncogene identified in a variety of human cancers including TNBCs [39] and is associated with poor clinical outcome [40]. High expression of *EGFR* is one of the traits of TNBC that may be targeted for treatment [41] as about 60% of basal-like BC over express *EGFR* [42] and *EGFR* inhibitors have been investigated in preclinical and clinical trials [43–47]. However, clinical trials have not yet demonstrated sufficient efficacy to warrant inclusion of *EGFR* inhibitors in standard treatment protocols. The DAMA model could potentially be used as a platform to study *Egfr* inhibitors in the future supported by immunohistochemical evidence (Fig. 1 Supplementary data). *Pgr* gene, which encodes the progesterone receptor, a key mediator of steroid hormone signaling [48] showed the third highest mutation frequency and was significantly enriched in the MTX-treated group compared to MTX-naïve tumors, This likely reflects treatment-driven clonal evolution, as chemotherapy can promote expansion of subclones harboring *Pgr* mutations [49]. Despite its high mutation frequency, immunohistochemistry revealed minimal (< 1%) progesterone receptor expression. Notably, *Pgr* upregulation has been reported in spontaneous adenocarcinomas, which may be relevant given the spontaneous origin of the DAMA tumor [50, 51].

The *Bcl2* gene, a key regulator of apoptosis [52] also had a high mutation frequency across all tumor samples. We hypothesize that this elevated mutation burden underscores the aggressive proliferative nature of the DAMA tumor, as dysregulation of apoptotic pathways is often associated with enhanced tumor survival and resistance to cell death [52]. *Bcl2*-directed therapies are emerging as promising strategies for treating TNBC as shown in preclinical studies [53, 54], and the DAMA tumor model has potential to be used to study *Bcl2* directed therapies in future applications.

Following *Bcl2*, the *Erbb3* gene, part of the epidermal growth factor receptor (*Egfr*) family exhibited a notably high mutation frequency across all tumor samples. This finding suggests a potential role for dysregulated *Erbb3* signaling in the progression of DAMA, consistent with its established involvement in promoting cell proliferation and mediating resistance to therapies targeting other *Egfr* family members [55]. The *Myc* gene, a key regulator of cellular growth, proliferation, metabolism, differentiation, and apoptosis [56, 57], exhibited a substantial mutation burden across all tumor samples analyzed with gene mutation frequency significantly increased in the MTX treated group compared to the MTX naïve group. The DAMA model may be explored as an avenue to study *Myc* mutations in human breast cancer as research have shown that chemotherapy can increase *Myc* mutations or dysregulation in BC by promoting resistant cell clones [58], causing DNA damage that leads to genomic instability [59], altering the tumor microenvironment to activate *Myc* pathways, and triggering epigenetic changes that boost *Myc* expression especially in aggressive subtypes like TNBC [60]. While no *Myc* inhibitors are yet approved for TNBC treatment, preclinical data strongly support their development [61–63].

Phosphatase and tensin homolog (*Pten*), a tumor suppressor gene frequently mutated in TNBC, negatively regulates the PI3K/Akt/mTOR pathway [64]and was found to be fairly mutated in DAMA tumor samples, suggesting a role in DAMA tumor progression. *Erbb4* mutations were minimal, consistent with its limited expression in breast cancer and its known pro-apoptotic and antiproliferative effects, including inhibition of *Erbb2* signaling [65]. *Esr1* (estrogen receptor alpha) also showed low mutational frequency, which may align with the hormone receptor negativity in TNBC and supported by immunohistochemical evidence of limited ERα protein expression in DAMA tumors.

Interestingly, several well-known oncogenes and tumor suppressors genes, including *Trp53*, *Kras*, *Bax* and *Erbb2* demonstrated remarkably low mutation frequencies, this may be attributed to the relatively smaller size of these genes in the DAMA tumor, which reduces the likelihood of accumulating mutations. However, in our study, we observed that the *Kras* gene mutation frequency significantly increased in the MTX treated group compared to the MTX naïve group which is not representative of population-level inference. *Kras* mutations, though rare in breast cancer, can increase post-chemotherapy in genomically unstable cell lines [66]. *Kras* pathway activation contributes to acquired resistance by bypassing targeted therapies and sustaining tumor growth [67]. Chemotherapy pressure may select for or induce *Kras* mutations, promoting expansion of resistant clones, making *Kras* activity a potential functional marker of therapeutic resistance [68].

*Bax* gene mutation frequency significantly decreased in the MTX treated group compared to the MTX naïve group. The low mutation burden in *Erbb2* is particularly notable given its established role in BC [69]. This finding is consistent with our immunohistochemical data, which showed low HER2 protein expression, suggesting that *Erbb2*-driven oncogenesis is not a dominant feature in the DAMA tumor. *Bax*, a pro-apoptotic protein, is essential for initiating cell death, and its reduced expression has been linked to poor response to chemotherapy and shorter survival [70] and research have shown that chemotherapy may decrease *Bax* mutation or expression in BC through mechanisms that impair the apoptotic pathway [70]. Furthermore, the *Trp53* gene, a critical tumor suppressor frequently mutated in various cancers [71], also exhibited a low mutation frequency. This supports the classification of the DAMA tumor as a *Trp53* wild-type subtype, which may have implications for therapeutic targeting. It is important to note that interpretation of mutations in genes such as *Bcl2*, *Egfr*, and *Myc* is based on variant frequency alone, without supporting data on expression, signaling, or functional dependency. These observations should therefore be considered hypothesis-generating pending future studies.

Results of our target gene analysis indicate that tumor mutational profiles can change following chemotherapy, though not uniformly across all genes. This aligns with observations by Tan et al., who reported rapid alterations in *EGFR*, *PIK3CA*, and *KIT* mutations after a single chemotherapy cycle in BC patients [72]. These results highlight intra-tumoral heterogeneity and tumor evolution as significant challenges to effective treatment which are also observed in DAMA. MTX treatment led to significantly increased mutations in oncogenes such as *Pgr*, Kras, and *Myc* probably due to replication stress, impaired DNA repair, and selective pressure favoring aggressive clones [73]. Conversely, MTX treated tumors showed significantly lower mutation frequency in *Bax* and *Erbb2*, likely due to preserved genomic stability [74, 75]. This shows that DAMA model may be used in-vivo to study *Erbb2*-targeted therapies in *Erbb2*-low tumors and enhancing apoptosis in *Bax* -intact tumors with pro-apoptotic agents [74, 76]. These strategies highlight the importance of understanding mutational landscapes even in both treated and untreated tumors to guide precision oncology approaches.

In our DAMA tumor samples, normalized mutation frequencies (mutations per gene length) were higher (≈ 0.15–0.30%) in *Bcl2*, *Esr2*, *Egfr*, *Erbb3*, *Myc*, *Pgr*, and *Pten*, and lower (≈ 0.00–0.05%) in *Bax*, *Esr1*, *Erbb2*, *Erbb4*, *Trp53*, and *Kras*. Placing these observations against human breast cancer WGS, TNBC genomes typically show early *TP53* disruption, frequent *PTEN* alterations, and MYC activation, alongside elevated *EGFR* pathway activity [77]; *ERBB2* amplification instead defines HER2‑positive disease, and *ERBB3*/*ERBB4* usually contribute via heterodimerization/crosstalk rather than recurrent coding drivers [78], features that align with DAMA’s low *Erbb2*/ *Erbb4* and *Esr1* and higher *Egfr*,/*Erbb3*/*Pten*/*Myc* mutation loads, while also acknowledging that in TNBC many driver events are copy‑number/structural‑variant mediated rather than point‑mutation heavy [79, 80]. Across mouse model WGS, the MMTV‑Neu (rat *Erbb2* transgene) and MMTV‑PyMT systems show receptor tyrosine kinase pathway modulation and extracellular matrix proteins gene copy‑number changes (e.g., *Ptprh* mutations that regulate *Egfr*, and amplifications of *Col1a1*/*Chad* that promote metastasis) [81], providing potential mechanistic parallels for DAMA’s higher rat *Egfr*/*Erbb3* signal and involvement of *Pten*/*Myc*. In BRCA1/p53 genetically engineered mouse models, *Trp53* lesions occur early, with *Myc* amplification and *Rb1* loss [82], features that potentially mirror human TNBC. While *Kras* amplification can emerge in certain contexts; notably, *Kras* (G12D) is sufficient to increase metastatic propensity in treatment‑naïve mouse models [83] even though *KRAS* point mutations are uncommon in human breast primaries [84, 85], which is consistent with DAMA’s low rat *Kras* mutation frequency. For rat WGS, rat mammary tumor studies (e.g., γ‑irradiation models) already demonstrate recurrent copy‑number losses and allelic imbalance, echoing the copy‑number–centric architecture seen in human TNBC [51]. Taken together, DAMA’s non‑HER2, EGFR‑active and *Myc*/*Pten*‑engaged profile with low rat *Esr1*/*Erbb2*/*Trp53*/*Kras* mutation frequencies and higher rat *Bcl2* versus lower *Bax* fits within a basal‑like/TNBC‑leaning continuum observed across rat, mouse, and human WGS. This positions the model’s translational utility while motivating orthology‑based copy number and structural variant benchmarking to confirm convergence of DAMA pathways with human TNBC genomes.

We acknowledge several limitations in our study, including the relatively small sample size which may amplify individual variability and limit the robustness and generalizability of the findings, and the collection of tumor samples exclusively at the study endpoint, rather than at multiple time points throughout the experimental timeline. This limits our ability to assess temporal changes in DAMA tumor evolution which could be addressed by exploring biopsies or circulating tDNA. While DAMA is isogenic, which is advantageous given that there is no need for immunosuppression, it is important to acknowledge that it is ultimately not human and thus its clinical relevance must acknowledge this. A limitation of this study is that all tumors were derived from a single donor lineage, which may reduce biological variability and limit generalizability, tumors originated from a common inoculum and were implanted bilaterally. While this design introduces microenvironmental variability, it may reduce genetic diversity and inflate statistical significance. Future studies should include multiple independent donor passages to improve robustness. The absence of matched non‑tumor DNA is a key constraint however, we mitigated this by subtracting DA/OlaHsd germline variants from the HRDP resource, while HRDP-based subtraction cannot fully replace matched normal tissue, these revisions materially reduce strain-SNP confounding and strengthen the evidence that the retained variants reflect tumor-associated events. Another limitation of our study is that the subtype classification relied on IHC using human scoring thresholds without antibody validation or orthogonal confirmation. Negative ER, PR, and HER2 expression may therefore reflect assay limitations rather than true receptor absence. These findings should be interpreted with caution, and future studies should include species-specific antibody validation and complementary assays to confirm receptor status.

Our findings suggest DAMA tumor is a *Trp53* wild type, *Egfr* overexpressing, *Bcl2* driven tumor. Understanding the specific subtype and immune composition of breast cancer in the DAMA model is essential for several reasons. Firstly, it allows for more precise and targeted research. Secondly, it can enhance the predictive power of the model in preclinical studies, leading to more accurate assessments of potential therapies. Lastly, it can help in identifying novel therapeutic targets and strategies tailored to the specific characteristics of the subtype.

Through this detailed characterization, we hope to provide valuable insights that will not only advance the field of breast cancer research but also contribute to the development of more effective and personalized therapeutic approaches.

## Conclusion

In conclusion, our DAMA tumor model exhibits key molecular and phenotypic features for modelling TNBC. Notably, we observed infiltration of immune cells, and this tumor appears to be primarily driven by *Bcl2*, *Egfr* and possibly *Myc* dysregulation, implicating apoptotic resistance as a central mechanism in its pathogenesis. This model offers a valuable preclinical platform that recapitulates the complexity of human TNBC, making it highly suitable for in-depth investigations into tumor biology, therapeutic response, and drug toxicity. It has a high potential to be utilized for human TNBC model which enhances its relevance for translational research, particularly in the development and evaluation of targeted therapies and immunotherapeutic strategies.

### Study-Specific Approval

All animal experiments were approved by the Animal Ethics Committee at the University of Adelaide (M-2023-018) in accordance with National Health and Research Council (Australia) NHMRC guidelines for use of laboratory animals.

## Supplementary Information

Supplementary Material 1.

Supplementary Material 2.

## Data Availability

Publicly available in a repository: Raw WGS data deposited on the National center for Biotechnology Information portal (NCBI BioProject: PRJNA1406328 ).
