# Supplementary material for: Exploring the Molecular and Genomic Landscape of the Dark Agouti Rat Mammary Adenocarcinoma: Preliminary Insights for Triple-Negative Breast Cancer Modeling
Source: J Mammary Gland Biol Neoplasia. 2026 Apr 10;31(1):15. doi: 10.1007/s10911-026-09606-x (PMC13156104; doi:10.1007/s10911-026-09606-x)
Supplement: Supplementary file 2 — Supplementary Material 2. [file 10911_2026_9606_MOESM2_ESM.docx]

**Supplementary Data**

**Table 1**: Normality Assessment of Gene Mutation Frequencies Using the Shapiro-Wilk Test. *Includes W statistics, p-values, inferred distribution type, and eligibility for t-test.*

| **Gene** | **W Statistic** | **P-value** | **Distribution** | **Eligible for t-test** |
| --- | --- | --- | --- | --- |
| BAX | 1.0000 | 0.0878 | Normal | Yes |
| BCL2 | 0.8336 | 0.0039 | Non-normal | No |
| ESR1 | 0.8661 | 0.0059 | Non-normal | No |
| ESR2 | 0.8881 | 0.0061 | Non-normal | No |
| HER1 | 0.8701 | 0.0042 | Non-normal | No |
| HER2 | 1.0000 | 0.1237 | Normal | Yes |
| HER3 | 0.9202 | 0.0436 | Non-normal | No |
| HER4 | 0.9316 | 0.0104 | Non-normal | No |
| MYC | 0.8802 | 0.0054 | Non-normal | No |
| P53 | 0.9402 | 0.0259 | Non-normal | No |
| PGR | 0.8921 | 0.0012 | Non-normal | No |
| PTEN | 0.8577 | 0.0034 | Non-normal | No |
| RAS | 0.8830 | 0.0000 | Non-normal | No |

**
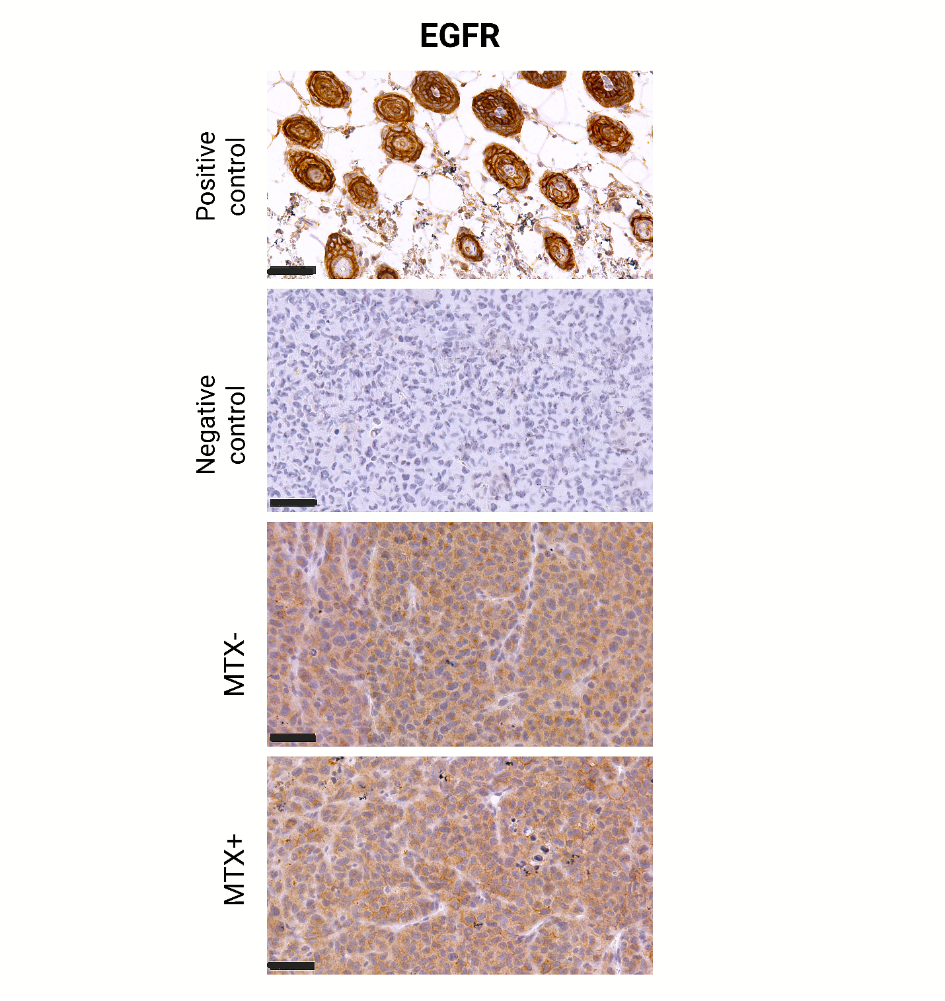
**

**Figure 1:** IHC staining for EGFR showing moderately intense membrane staining in DAMA tissue with a degree of intensity observed in >1% of tumor membrane. MTX- = MTX naïve, MTX+ = MTX treated. Positive control = mouse skin. Scale bar- 50µm.


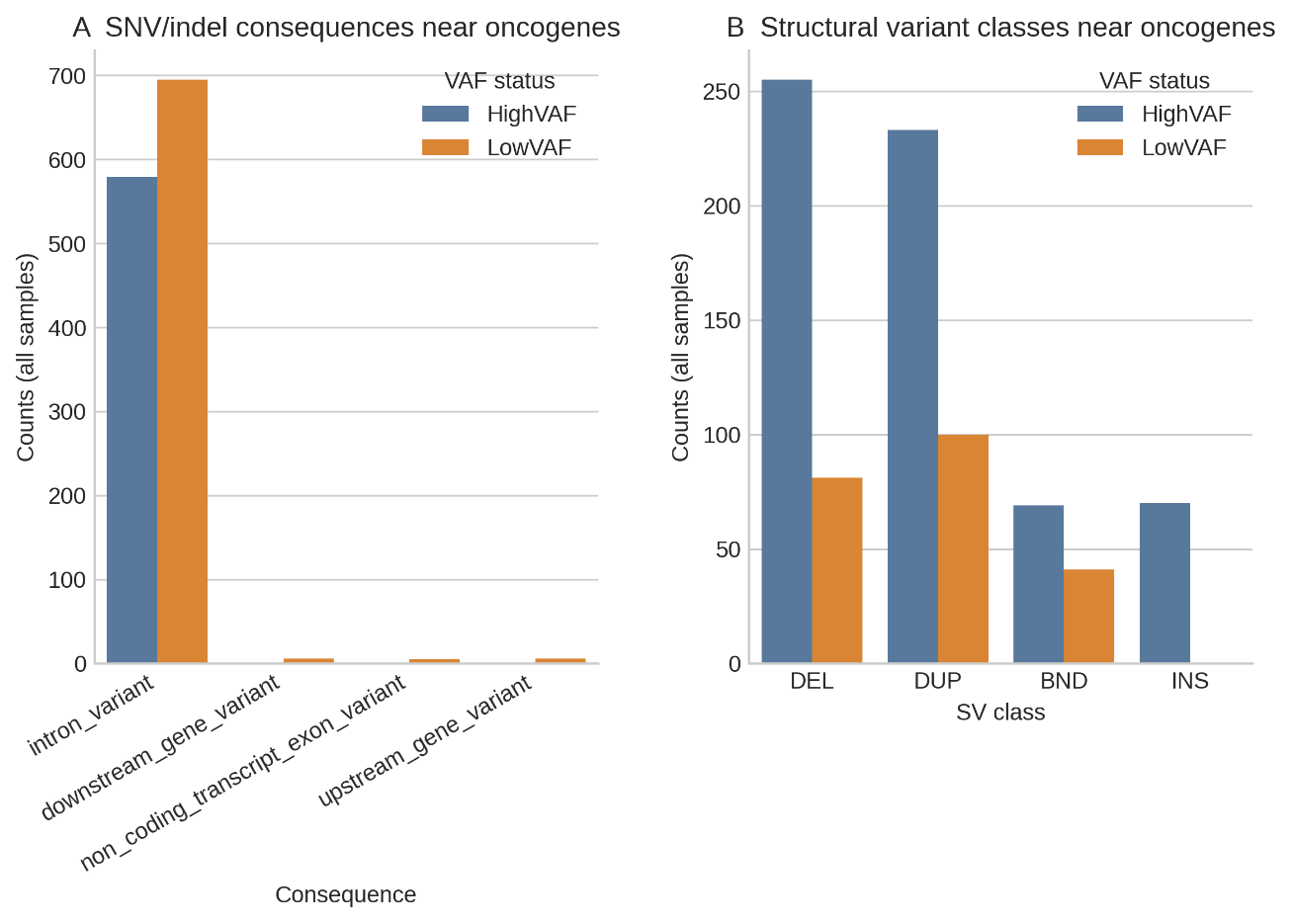


**Figure 2**: Oncogene‑proximal variant landscape (A): bar plots of SNV/indel consequences by VAF status. (B): bar plots of SV classes by VAF status, both aggregated across all samples.

**
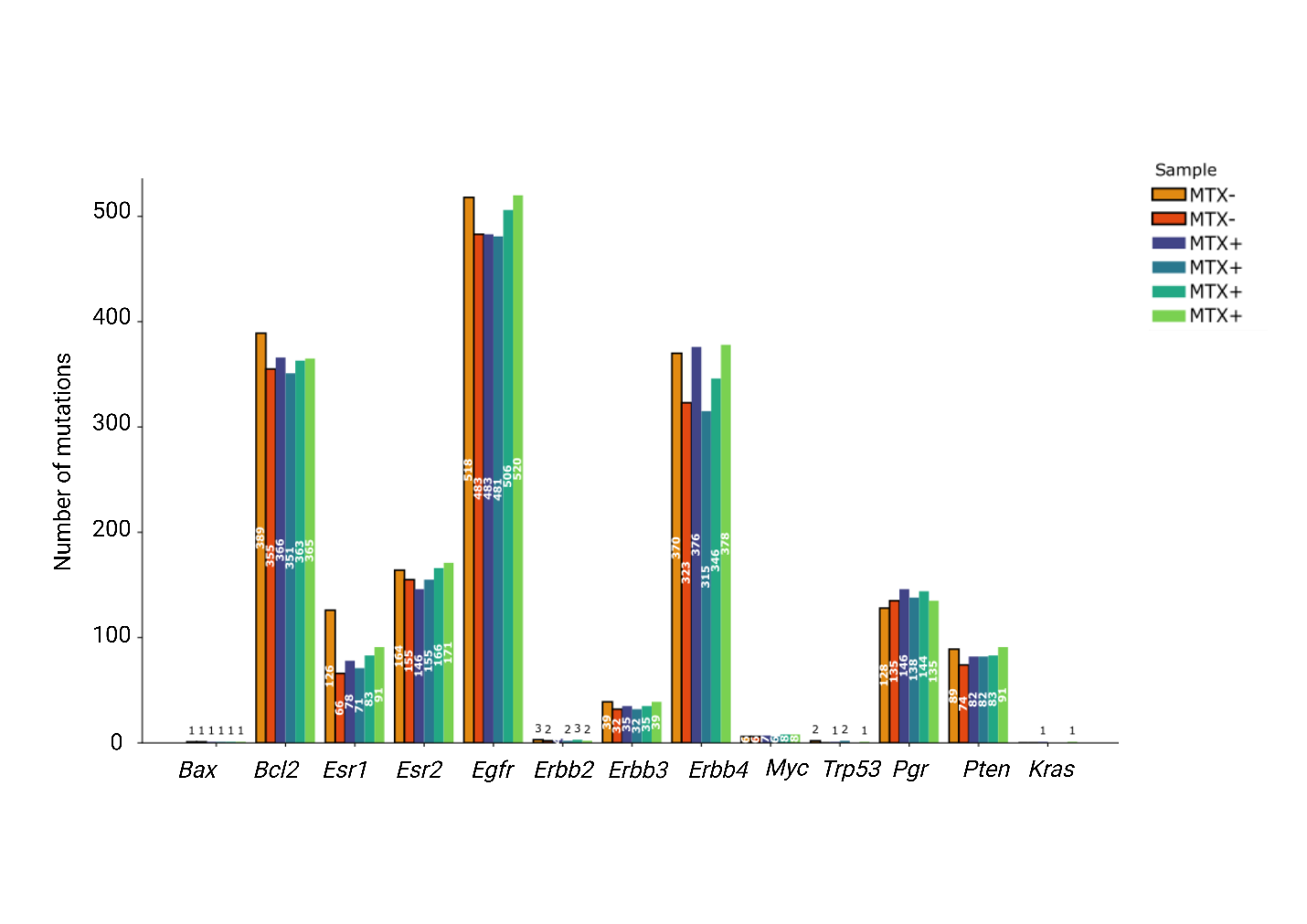
**

**Figure 3:** Gene mutation count across 13 cancer-related genes in DAMA tumor samples. Bar graph shows the percentage of mutations detected in each of the analyzed cancer-related genes (*Bax, Bcl2, Esr1, Esr2, Egfr, Erbb2, Erbb3, Erbb4, Myc, Trp53, Pgr, Pten, and Kras*), normalized by gene length, across all six samples. Values shown above each bar represent the mutation frequency as a percentage relative to the length of each gene.
